# An enhanced lure for eastern populations of the North American spruce beetle, *Dendroctonus rufipennis* Kirby (Coleoptera: Curculionidae)

**DOI:** 10.1093/jee/toae125

**Published:** 2024-06-02

**Authors:** Deepa S Pureswaran, Rylee Isitt, Dezene P W Huber

**Affiliations:** Natural Resources Canada, Canadian Forest Service-Atlantic Forestry Centre, Fredericton, NB, Canada; Natural Resources Canada, Canadian Forest Service-Atlantic Forestry Centre, Fredericton, NB, Canada; Faculty of Environment, University of Northern British Columbia, Prince George, BC, Canada

**Keywords:** Bark beetles, semiochemicals, new attractive lure, regional variation, *Dendroctonus simplex*, *Thanasimus dubius*

## Abstract

Regional variation in pheromone production and response has practical implications for the use of semiochemical lures to monitor and control bark beetle populations. We tested 4 lure formulations including 2 new formulations that reflect the pheromone production profiles of western and eastern populations of spruce beetles, *Dendroctonus rufipennis* Kirby (Coleoptera: Curculionidae), as well as 2 commercially available formulations (current Rocky Mountain lure and current Atlantic lure), in 2 locations in New Brunswick, Canada. In 2 separate years, the new eastern lure containing seudenol, MCOL, and spruce terpenes captured 4 times (2021) and 11 times (2022) more spruce beetles than the current Atlantic lure that consisted of frontalin, seudenol, and spruce terpenes. In 2021, we also captured more eastern larch beetles, *Dendroctonus simplex* LeConte (Coleoptera: Curculionidae), with the new eastern lure, whereas in 2022, we captured the most *D. simplex* with the current Atlantic lure, suggesting that more research is needed on *D. simplex* pheromone production and response across its range. The bark beetle predator, *Thanasimus dubius* (Fabr.; Coleoptera: Cleridae), did not respond well to the new eastern blend that lacks frontalin, suggesting that response to frontalin is important in finding prey and might be conserved in predator populations. The reduced trap catch of *T. dubius* to the enhanced lure is beneficial because it does not inhibit natural population control by removing predators from the community. Our study reveals an improved trap lure for eastern populations of spruce beetles and highlights gaps and research needs in bark beetle pheromone ecology.

## Graphical Abstract

**Graphical Abstract:**
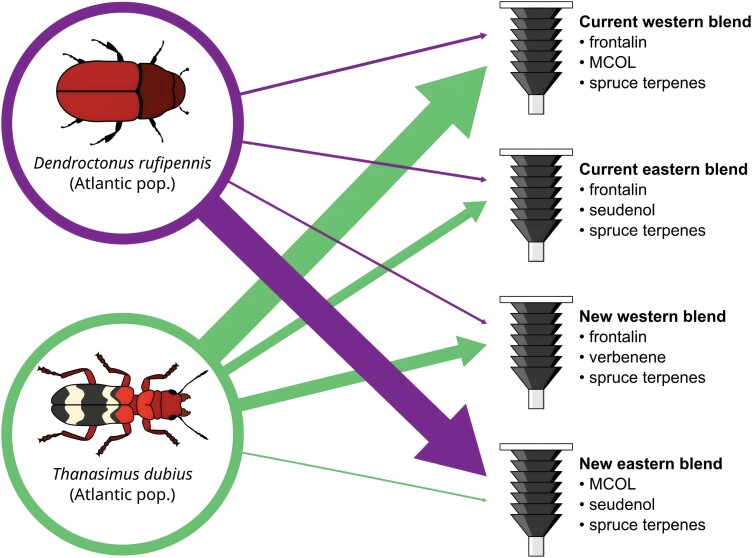

## Introduction

Populations of the spruce beetle, *Dendroctonus rufipennis* Kirby (Coleoptera: Curculionidae), are trans-continental in distribution extending from Newfoundland in the east, to Alaska in the west, and south from Alaska to high-elevation spruce forests in northern Arizona (Furniss and Carolin 1977, Wood 1982), covering a latitudinal gradient between 32°N and 65°N (Bentz and Jönsson 2015). Spruce beetle outbreaks have been widespread in recent decades, affecting 2 million hectares of forest in Alaska in the 1980s and 1990s (Werner et al. 2006), as well as the western United States and Canada from the 2000s to the present day (Negron and Popp 2018, BC FLNRORD 2020, Bleiker et al. 2021). Spruce beetles usually attack fallen or recently dead trees, but when populations build up to outbreak levels, beetles can kill standing trees (Schmid and Frye 1977, Werner and Holsten 1983). More recently, outbreaks are occurring in eastern Canada, including the Fundy National Park in New Brunswick (Pureswaran pers. obs. 2022; NB DNR Pest Report 2023) resulting in tree mortality. Population growth and tree mortality have been attributed to warmer summer temperatures that facilitate univoltinism in otherwise semivoltine populations (Werner and Holsten 1985, Hansen and Bentz 2003).

Like other bark beetle species that are lethal to their host trees, spruce beetles use a pheromone-mediated mass attack strategy to aggregate in large numbers to overcome host defenses (Theis and Lerdau 2003, Franceschi et al. 2005). Females are the first attacking sex (Wood 1982) and begin producing aggregation pheromones on entering the tree (Isitt et al. 2018). They are joined by males who also contribute to pheromone production (Isitt et al. 2018). Hundreds of beetles are thereby attracted to the tree and colonize in synchrony to successfully reproduce (Raffa and Berryman 1983). Once an optimum population density is reached, both sexes produce antiaggregation pheromones that terminate attraction, preventing overcrowding and high competition, and switch the attack to neighboring trees (Werner and Holsten 1995). In spruce beetles, frontalin (1,5-dimethyl-6,8-dioxabicyclo[3.2.1.]octane; Dyer 1973, Gries et al. 1988), MCOL (1-methyl-2-cyclohexen-1-ol; Borden et al. 1996), seudenol (3-methyl-2-cyclohexen-1-ol; Vité et al. 1972, Furniss et al. 1976) and verbenene (4-methylene-6,6-dimethylbicyclo[3.1.1.]hept-2-ene; Gries et al. 1992) have been identified as aggregation pheromone components. In combination with aggregation pheromones, host terpenes released by trees under stress facilitate beetle orientation to infested spots in stands, synergizing mass attacks on trees on which attack has begun (Pureswaran and Borden 2005). MCH or 3-methyl-2-cyclohexen-1-one is the only antiaggregation pheromone that is currently known to repel spruce beetles (Rudinsky et al. 1974).

Since the pheromone-mediated mass attack is critical to the colonization success of bark beetles, synthetic pheromone lures in combination with host tree volatiles are useful tools to monitor and control bark beetle populations (Pitman 1969, Borden 1995). Aggregation pheromones such as frontalin are used to bait standing trap trees in forests to attract beetles into attacking baited trees, with subsequent removal and milling or burning of these beetle-filled trees as a method of population control (Dyer 1975, Dyer and Safranyik 1977, Hodgkinson 1985). Conversely, MCH, the antiaggregation pheromone, is used to protect live spruce trees by repelling beetles and reducing the number of beetles drawn to standing trees (Holsten et al. 2003, Audley et al. 2022). Traps baited with aggregation pheromones and host volatiles are also used to monitor population trends to inform forest managers of insect activity and population densities, particularly to detect incipient and rising populations (Negron and Popp 2018). However, natural variation in the production of and response to different aggregation pheromone blends among regional populations reduces the efficacy of generalized lure formulations deployed in traps (Borden et al 1996). Therefore, trap catch numbers when surveying for spruce beetles or conducting experiments are often low (Pureswaran and Borden 2004, Isitt et al. 2023). Of the 4 known aggregation pheromone components, previous research reported that frontalin dominated in western Canada (Borden et al. 1996) and seudenol in the east (Ryall et al. 2013). The commercial pheromone lure for traps currently incorporates frontalin, MCOL, and spruce terpenes for western populations and frontalin, seudenol, and spruce terpenes in the east (Synergy Semiochemicals Corporation). Commercial tree baits consist of frontalin alone, regardless of region (Synergy Semiochemicals Corporation).

Regional variation in pheromone production and response could be attributed to the geographic isolation of beetle populations (Lanier et al. 1980, Horn et al. 2006), interactions with competitors, cooperators, or predators (Raffa and Dahlsten 1995, Raffa 2001) or regional variation of host volatile profiles (Pureswaran et al. 2004, Taft et al. 2015). In recent studies, we found variation at both large and small spatial scales in pheromone production profiles of the spruce beetle. Isitt et al. (2020) identified 4 aggregation blend ratios produced by beetles: (1) the **‘F’ profile** was dominated by frontalin and occurred in BC; (2) the **‘FV’ profile** consisted of approximately equal parts of frontalin and verbenene and was detected in Rocky Mountain House, AB; (3) the **‘M’ profile** consisting of a 4:1 ratio of MCOL: seudenol was present in varying degrees in all populations sampled, but most prominently, in eastern Canada; and (4) the **‘S’ profile** dominated by seudenol was detected in populations east of the Rockies and not in BC.

We tested 4 lure formulations, 2 new formulations that reflected the above profiles for western and eastern populations (FV and M) as well as 2 current commercially available formulations (current Rocky Mountain lure and current Atlantic lure) in 2 locations in New Brunswick, Canada, where spruce beetle populations are currently present (NB DNR 2023). We predicted that eastern populations would respond in higher numbers to the ‘**M profile**’ containing both MCOL and seudenol that corresponds to pheromone production profiles in eastern populations.

## Materials and Methods

### Study Site and Beetle Trapping

We conducted field trapping experiments in mature spruce-dominated forests in Fundy National Park (45.566°N, −64.983°W) and Memramcook (46.042°N, −64.560°W) in New Brunswick, Canada in 2021 and 2022, respectively. Tree species in Fundy were 70% red spruce, 10% balsam fir, 10% yellow birch, and 10% white birch. In Memramcook, there was 60% white spruce, 20% eastern larch, 10% white pine, and 10% other softwood species. Spruce beetle populations were visibly present, with spruce trees containing pitch tubes and beetle entrance holes throughout the bole. Twelve-unit multiple-funnel traps (Lindgren 1983) were suspended by a rope between pairs of trees with traps spaced at least 15 meters from each other. We set up traps in randomized complete blocks, 10 blocks in each site, with the following treatments (1) current Rocky Mountain lure (frontalin, MCOL, spruce terpenes); (2) current Atlantic lure (frontalin, seudenol, spruce terpenes); (3) new western lure (frontalin, verbenene, and spruce terpenes; **FV profile)**; and (4) new eastern lure (MCOL, seudenol, and spruce terpenes; **M profile)**. Lure components were spruce terpenes released at 120 to130 mg/d at 25°C; frontalin released at 1.5 mg/d at 20°C; MCOL released at 2 to 4 mg/d at 25°C; seudenol released at 2 to 4 mg/d at 25°C and verbenene released at 1 to 2 mg/d at 25°C. All semiochemicals were >95% pure and were obtained from (Synergy Semiochemicals Corporation, Delta, British Columbia, Canada). Lures were suspended on the outside of the traps on the 4th, 6th, and 8th funnels from the top. We filled trap cups with a solution of table salt (NaCl) and water to drown incoming beetles. Trap catches for the 2021 Fundy experiment were collected weekly for a total of 8 collections between May 31 and July 26 (with no collection on July 19). Trap catches for the 2022 Memramcook experiment were collected every 2 wk for a total of 7 collections between June 6 and August 29. We counted captured beetles of *D. rufipennis*, *D. simplex* LeConte (Coleoptera: Curculionidae) and *Thanasimus dubius* (Fabr.; Coleoptera: Cleridae) for each collection date. We counted males and females separately for *D. rufipennis* and *D. simplex*, but used total species counts for *T. dubius*.

### Statistical Analyses

We summed beetle counts across all collection dates per trap. For the Memramcook experiment in 2022, 2 traps fell over during the field experiment, resulting in 2 trap collections (out of 280) that were not collected. To prevent this from introducing a downward bias into the counts of beetles for the affected trap treatments, we excluded the collections from all other traps in the affected blocks and collection dates (an additional 6 trap collections). To determine if the different semiochemical treatments resulted in different counts of captured beetles for each species and sex, we modeled beetle counts using Poisson (square-root link) generalized linear mixed models with semiochemical treatment as a fixed effect and block as a random effect. We compared the fit of these models to reduced models (lacking the treatment effect) using likelihood ratio tests. If these tests indicated significant differences (α = 0.05) between the full and reduced models (evidence for an effect of semiochemical treatment), we used post hoc contrasts of estimated marginal means (using Tukey’s method for correcting for multiple comparisons) to compare the trap catches between all pairwise combinations of semiochemical treatments. All statistical analyses were conducted in R 4.3.1 (R Core Team 2023), with the ‘lme4’ package for generalized linear mixed models (Bates et al. 2015) and the ‘emmeans’ package for post hoc tests (Lenth 2023).

## Results

**Figure 1.**
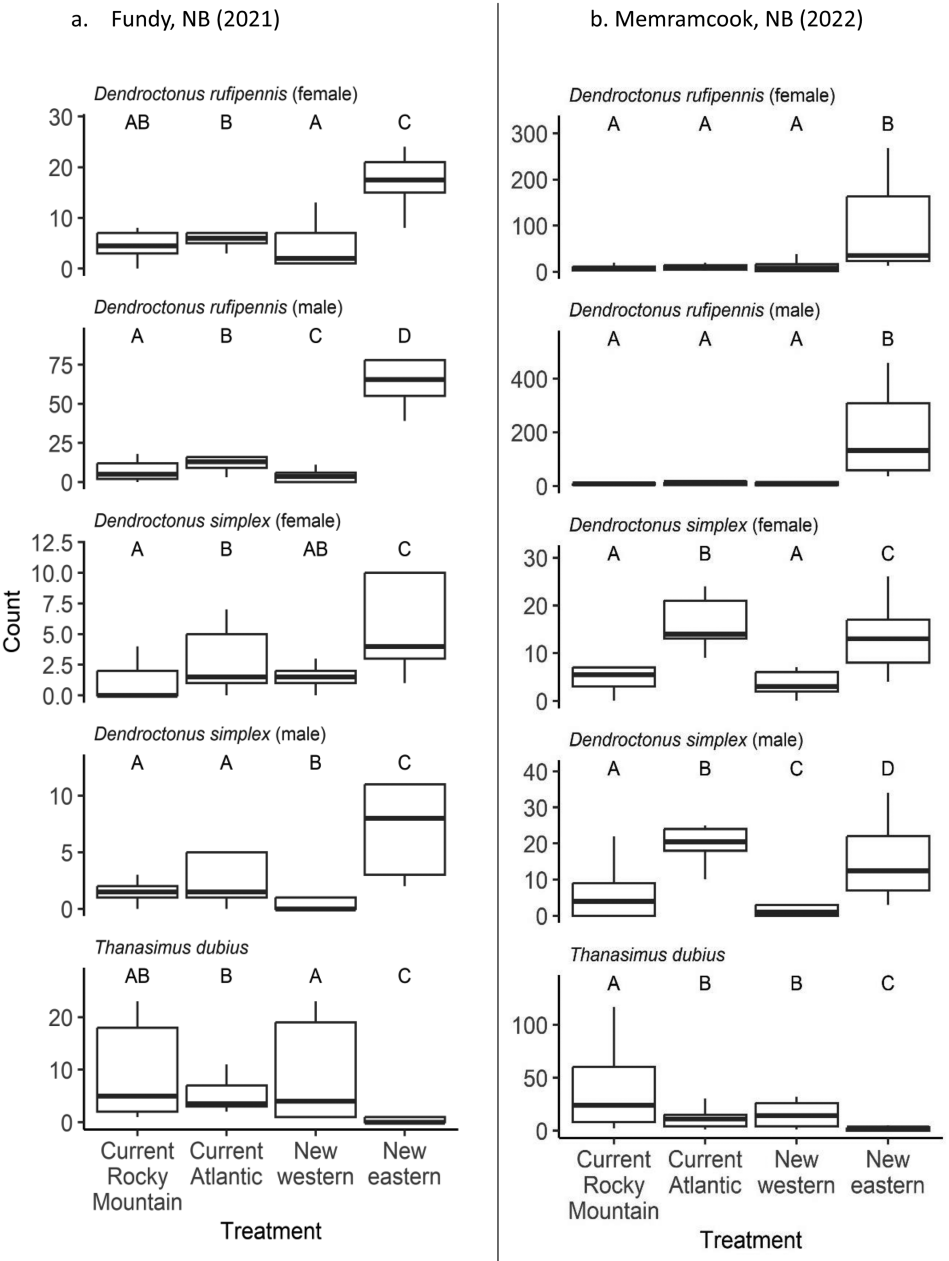
Counts of bark beetles and clerid predators captured in multiple-funnel traps amended with a variety of commercial and experimental lures for *Dendroctonus rufipennis* (*n* = 10 for each treatment). Different letter designations above boxplots indicate statistically significant differences (within sub-plots) in pairwise Tukey’s tests of marginal means following generalized linear mixed effects models (α = 0.05). Outliers are omitted from boxplots for clarity. Horizontal line within each boxplot represents the median value. a. Fundy, NB (2021). b. Memramcook, NB (2022)

The semiochemical treatment was a significant predictor of the count of trapped insects in all models, regardless of study year, insect species, or sex (likelihood ratio tests: lowest *X*^2^ = 69.0, *df* = 3; *P* < 0.001; Table 1). In post hoc tests, the new eastern lure was the only treatment that consistently differed from the others (Figure 1). When compared to the current Atlantic *D. rufipennis* lure formulation, the new eastern lure (containing MCOL instead of frontalin) captured 4 times as many *D. rufipennis* in Fundy National Park in 2021 (female *z-ratio* = −6.96; *P* < 0.0001; male *z-ratio* = −21.8; *P* < 0.0001), and 11 times as many *D. rufipennis* in Memramcook in 2022 (female *z-ratio* = −23.6; *P* < 0.0001; male *z-ratio* = −44.0; *P* < 0.0001). The new eastern lure caught less than one-fifth the number of *T. dubius* as the current Atlantic lure in both Fundy National Park (*z-ratio* = 6.95; *P* < 0.001) and Memramcook (*z-ratio* = 8.88; *P* < 0.0001). We also captured the eastern larch beetle, *D. simplex*, in both years at our sites. In 2021, the new eastern lure captured more *D. simplex* of both sexes than the current Atlantic lure (female *z-ratio* = −4.33; *P* = 0.0001; male *z-ratio* = -10.3; *P* < 0.0001; Fig. 1a). However, in 2022, at a site ~100 km away, the results were reversed, with the current Atlantic lure capturing more *D. simplex* of both sexes than the new eastern lure (female *z-ratio* = 2.70; *P* = 0.035; male *z-ratio* = 4.58; *P* < 0.0001; Fig. 1b).

**Table 1.** Total numbers of bark beetles and clerid predators captured in multiple-funnel traps amended with a variety of commercial and experimental lures for *Dendroctonus rufipennis*. Statistics (*χ*^*2*^ and *p*) are the results of likelihood ratio tests between linear models including a fixed effect of semiochemical treatment and reduced models without

		Lure formulation					
Experiment	Species and sex	Current Rocky Mountain	Current Atlantic	New western	New eastern	*χ* ^*2*^ (*df* = 3)	*P*
Fundy, NB (2021)	*Dendroctonus rufipennis*						
	Female	47	76	43	185	133	<0.001
	Male	101	147	37	759	1,092	<0.001
	*Dendroctonus simplex*						
	Female	10	35	18	84	69.0	<0.001
	Male	15	42	5	241	266	<0.001
	*Thanasimus dubius*	86	62	130	11	94.4	<0.001
Memramcook, NB (2022)	*Dendroctonus rufipennis*						
	Female	156	113	111	860	841	<0.001
	Male	124	132	107	1,989	3,139	<0.001
	*Dendroctonus simplex*						
	Female	70	177	36	131	117	<0.001
	Male	57	239	13	152	283	<0.001
	*Thanasimus dubius*	353	115	151	21	300	<0.001

Significant results indicate that the different lure formulations attracted different numbers of bark beetles or predators.

## Discussion

Our results were consistent with our prediction that the new eastern lure consisting of MCOL, seudenol, and spruce terpenes would be more attractive to eastern spruce beetle populations than the current commercially available Atlantic lure consisting of frontalin, seudenol, and spruce terpenes (Fig. 1a,b). This finding suggests that regional variation in pheromone response reflects pheromone production profiles and highlights the importance of systematic approaches to elucidating quantitative and qualitative variation in production and response profiles of semiochemicals for pests with large species distribution areas. Eastern populations of spruce beetle females produced almost exclusively MCOL and seudenol before they were joined by males (Isitt et al. 2020), suggesting that this combination of pheromone components was likely to be highly attractive to both males and females if beetles were to successfully colonize a tree. We only tested racemic (+) versions of the compounds that are commercially available. However, enantiomeric ratios of optically active compounds produced by beetles may be particularly important in enhancing attraction to traps. Beetles produce 95% (−)-frontalin, 90% (+)-verbenene, 70% (+)-MCOL, and 80% (+)-seudenol in most locations (Isitt et al. 2020). The effect of using enantio-specific lures in traps is hitherto underexplored. Our results suggest that fine-scale modifications to pheromone lures could increase attraction efficacy to both traps and trap trees across the range of the spruce beetle. In western Canada, Setter and Borden (1999) showed that in northern BC, MCOL enhanced the attraction of both sexes to traps baited with frontalin. There was no enantio-specific preference for MCOL in northern BC, southeastern BC, and northern AB. Enantio-specific populations occurred in southern BC and Alaska with beetles responding only to (+)-MCOL. However, on baited trap trees, neither MCOL nor seudenol increased the attraction of frontalin.

Interpopulation differences in pheromone production and response have been documented in several species of bark beetles including the western pine beetle, *Dendroctonus brevicomis* LeConte (Coleoptera: Curculionidae; Pureswaran et al. 2008, 2016a, b), the southern pine beetle, *Dendroctonus frontalis* Zimmermann (Coleoptera: Curculionidae; Berisford et al. 1990) and the pine engraver, *Ips pini* Say (Coleoptera: Curculionidae; Lanier et al. 1980, Miller et al. 1989, 1997). In some instances, geographic variation in pheromone production has been attributed to interspecific interactions between sympatric species.

Western pine beetle populations were originally classified as 2 separate species—*D. brevicomis* west of the Great Basin and *D. barberi* Hopkins to the east—were synonymized by Wood (1963) and then separated again (Valerio-Mendoza et al. 2019) following morphological, genetic, and chemical ecological studies. Chemical ecological divergence included high production of *endo*-brevicomin by females in Arizona, compared to populations in California and Oregon that produced the *exo*-isomer (Pureswaran et al. 2016a, b). Similar patterns were observed in their response to pheromone-baited traps (Bedard et al. 1980, Pureswaran et al. 2016b). Geographic variation in the production of *endo*-brevicomin in the Arizona population was attributed to pheromone convergence with a congener, the southern pine beetle, as it is the only region where both species occur in sympatry and can co-attack the same *Pinus* spp. mediated by the same pheromone components (Pureswaran et al. 2016a).

In pine engraver beetles, populations in California produced and responded predominantly to (R)-(−)-ipsdienol (Birch et al. 1980, Seybold 1992) whereas New York populations produced equal amounts of (R)-(+) and (S)-(−)-ipsdienol (Lanier et al. 1980, Teale and Lanier 1991). In addition, lanierone synergized the attraction of ipsdienol in New York and Wisconsin, but not in western populations (Miller et al. 1997). Western populations of *I. pini* co-occur with the California 5-spined ips, *Ips paraconfusus* (Miller et al. 1997) that produces (4S)-(+)-ipsdienol (Silverstein et al. 1966, Fish et al. 1984) and production and response of the antipode by *I. pini* helps to maintain reproductive isolation in sympatry. In spruce beetles, 2 genetically distinct, sympatric clades have been documented (Maroja et al. 2007). However, whether the pheromone variation we observe among populations reflects genetic variation is not yet known.

Congeners of spruce beetle on the same host tree species include *Dendroctonus punctatus* LeConte (Coleoptera: Curculionidae) that infests the lower bole of spruce (Furniss 1995), but for which no pheromones have been identified. However, other *Dendroctonus* spp. do occur in the same forest stands when their host species are sympatric with spruce. For example, spruce beetle frequently cooccurs with Douglas-fir beetle, *Dendroctonus pseudotsugae* Hopkins (Coleoptera: Curculionidae) in western North America and the 2 species share frontalin, seudenol, and MCOL as components of their pheromone blends (Gries et al. 1992, Lindgren 1992). In our sites in eastern Canada, spruce beetle cooccurs with the eastern larch beetle, *D. simplex*. Frontalin and seudenol are shared pheromone components between spruce beetles and eastern larch beetles reported in Alaskan populations (Baker et al. 1977). Pheromone blends produced by eastern populations of *D. simplex* and their fine-scale variation are not known. However, our experiments show that the new eastern lure containing seudenol and MCOL captured the most beetles in 2021 while the current Atlantic lure containing frontalin and seudenol was most attractive in 2022 at a site ~100 km away (Fig. 1, Table 1). It is possible that this difference in response of *D. simplex* between the 2 yr could be due to fine-scale geographic variation in pheromone attraction. Elucidating the regional pheromone profiles of *D. simplex* across its range would therefore be a worthwhile endeavor.

*Thanasimus dubius* is a generalist clerid predator of bark beetles that is widespread across North America (Furniss and Carolin 1977). They use conifer volatiles and bark beetle pheromones to orient towards prey they capture and are therefore attracted to traps containing ethanol, conifer volatiles, and bark beetle pheromone components (Miller 2023). *T. dubius* attacks all *Ips* spp. and *Dendroctonus* spp. across its range from the Gulf of Mexico to Alaska to eastern North America including Atlantic Canada (Reeve et al. 2009). It is important in driving the population dynamics of bark beetles (Reeve 1997, Turchin 1999, Aukema and Raffa 2002). We found that *T. dubius* responded poorly to the new eastern lure that did not contain frontalin (Fig. 1, Table 1). Frontalin is strongly attractive to *T. dubius* (Reeve et al. 2009) as it is a pheromone of several *Dendroctonus* spp. including *D. ponderosae*, *D. rufipennis*, *D. frontalis*, *D. pseudotsugae*, *D. simplex*, *D. terebrans* (Olivier), and *D. valens* LeConte (Symonds and Gitau-Clarke 2016). It appears that *T. dubius*’ response to frontalin is conserved even in regions where frontalin is not the dominant pheromone component of *D. rufipennis*. Reeve et al. (2009) examined geographic variation in pheromone response of *T. dubius* to prey pheromones to determine if there was regional variation in their response to prey pheromones. They found that the predator decreased its response to *Ips* pheromones at sites where there were outbreaks of *D. frontalis,* a frontalin-emitter. They also found that *T. dubius* exhibited a strong response to frontalin even when prey such as *D. frontalis* or *D. rufipennis* were at low densities or apparently absent, suggesting that there could be gene flow among *T. dubius* populations that might maintain predator response to frontalin across the range of a variety of *Dendroctonus* spp. From a pest management perspective, the new eastern lure is highly attractive to spruce beetle, significantly improving the current Atlantic lure, while allowing its predators to exert natural enemy control instead of capturing them in traps.

## Future Research

We identified several gaps in bark beetle pheromone ecology that merit further research. Variation in pheromone production and response across the range of bark beetle species, variation in production by individual beetles during the host colonization process, and the correlation of such variation with genetic variation would help identify cryptic species. Considering the huge geographic range, the variety of hosts that are used, and the variation in pheromone production and response, it may be possible that some spruce beetle populations are in fact cryptic species, similar to the situation with *D. brevicomis* and *D. barberi* before they were split in 2019. Geographic variation in *D. pseudotsugae* and *D. simplex* chemical ecology across their respective ranges is also not known. It is also important to quantify differences in host tree chemistry across the vast geographic ranges of conifer species and to test for correlation with pheromone production in *Dendroctonus* spp. Further tests to determine whether lure concentrations that closely reflect beetle-produced concentrations are more effective, would be useful. While biosynthetic pathways for frontalin and verbenene in *D. rufipennis* have been proposed (Barkawi et al. 2003 and, Blomquist et al. 2010), no pathways have been identified for MCOL, seudenol, or MCH biosynthesis. Understanding pheromone biosynthesis will help identify genes and precursor chemicals (host-derived or otherwise) whose variation may explain and predict similar variations in pheromone production.

## Data Availability

Data from this study is available from the figshare digital repository: DOI:10.6084/m9.figshare.25848601 Pureswaran 2024.
